# Integrating qualitative interviews in drug development and the use of qualitative evidence in product labelling and health technology assessments: a review

**DOI:** 10.3389/fmed.2023.1197529

**Published:** 2023-06-20

**Authors:** Anne-Sophie Michel, Paul Kamudoni, Alexia Marrel, Rocco Adiutori, Céline Desvignes-Gleizes, Sally Lanar, Peter Schache, Erica Spies, Josephine Park

**Affiliations:** ^1^ICON Plc, Patient Centered Outcomes, Lyon, France; ^2^Merck KgaA, Clinical Measurement Sciences, Global Research and Development, Healthcare, Darmstadt, Germany; ^3^Mapi Research Trust, Lyon, France; ^4^LAIFE Reply, Frankfurt, Germany; ^5^EMD Serono Research and Development Institute Inc., A Business of Merck KGaA, Patient Centered Outcomes Research, Global Research and Development Healthcare, Darmstadt, Germany

**Keywords:** qualitative research and analysis, interview, patient-focused drug development, chronic disease, mixed-method approaches, clinical trial

## Abstract

**Objective:**

Including qualitative research in clinical trial design is an innovative approach to understanding patients’ perspective and incorporate the patient’s voice in all stages of drug development and evaluation. This review aims to explore current practices, lessons learned from the literature, as well as how qualitative interviews are considered by health authorities for marketing authorization and reimbursement.

**Methods:**

A targeted literature review of Medline and Embase databases was conducted in February 2022 to identify publications on qualitative methods embedded in clinical trial of pharmaceutical products. An additional search of guidelines and labeling claims of approved products regarding qualitative research was performed in various sources of grey literature.

**Results:**

From the 24 publications and nine documents reviewed, we identified the research questions addressed with qualitative methods during clinical trials (e.g., change in quality of life, symptoms assessment, treatment benefit), preferred data collection methods (e.g., interviews), and data collection points (e.g., baseline and exit interviews). Moreover, the data from labels and HTAs demonstrate that qualitative data can play an important role in approval processes.

**Conclusion:**

The use of in-trial interviews is still emerging and is not yet common practice. Although the industry, scientific community, regulatory agencies and HTAs are showing an increasing interest in the use of evidence generated via in-trial interviews, guidance from regulators and HTAs would be helpful. Developing new methods and technologies to address the common challenges for such interviews is key to progress.

## 1. Introduction

**Figure 1:**
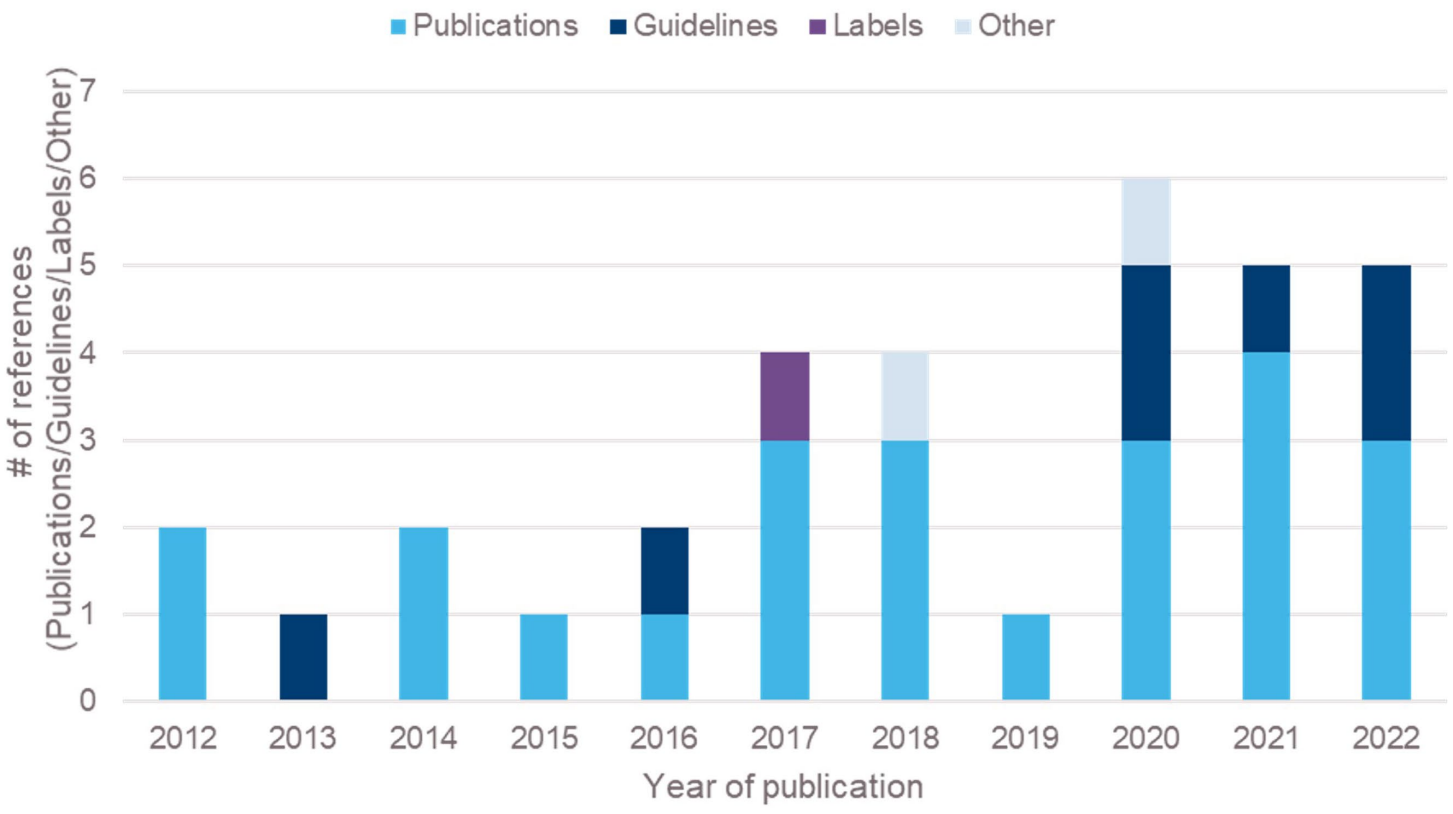
Overview of qualitative research embedded in clinical trials (2012–2022).

Patient-focused drug development (PFDD) aims to support systematic capture and meaningful incorporation of patients’ voices (experiences, perspectives, needs and priorities) into all stages of drug development and evaluation, and continues to be one of the innovations shaping drug development (1). Recent guidance on PFDD from the FDA suggests multiple research methods to understand what is important to patients, including qualitative, quantitative, or mixed methods research (MMR) (2). While qualitative research methods such as interviews and focus groups are used across many fields, within drug development these methods have been primarily used to support the generation of content validity evidence for patient-reported outcome (PRO) measures (3–5). The use of such methods to address other objectives within drug development, beyond the above, is relatively recent (6), and is on the increase. For example, peer-reviewed articles and guidelines around the use of in-trial interviews doubled between 2012 and 2022 (Figure 1).

In-trial interviews provides a platform to gain insights on the drug under investigation, e.g., providing an understanding of the patient’s experience of the treatment, insights on which specific symptoms or impacts change during a trial, clarity on the meaningfulness of such changes, and supporting the interpretation of other quantitative assessments in a trial, such as PRO measures (2, 7). Such interviews can be embedded within clinical trials and included as a trial objective or may be conducted as a stand-alone study in parallel to the clinical trial, recruiting participants from the trial.

Given the novelty of such approaches, we conducted a literature and guideline review to understand (1) the current practices in the implementation of patient interviews in drug development in terms of the type of questions addressed, the methodology employed and (2) how data generated from interviews are considered by health authorities for marketing authorization and reimbursement. We share the lessons learned from the literature and recommendations on the conduct of in-trial interviews.

## 2. Methods

A targeted literature review was performed to identify clinical trials integrating interviews, and regulatory guidelines and documents, and medicinal product labels including any mention of qualitative research.

**Figure 2:**
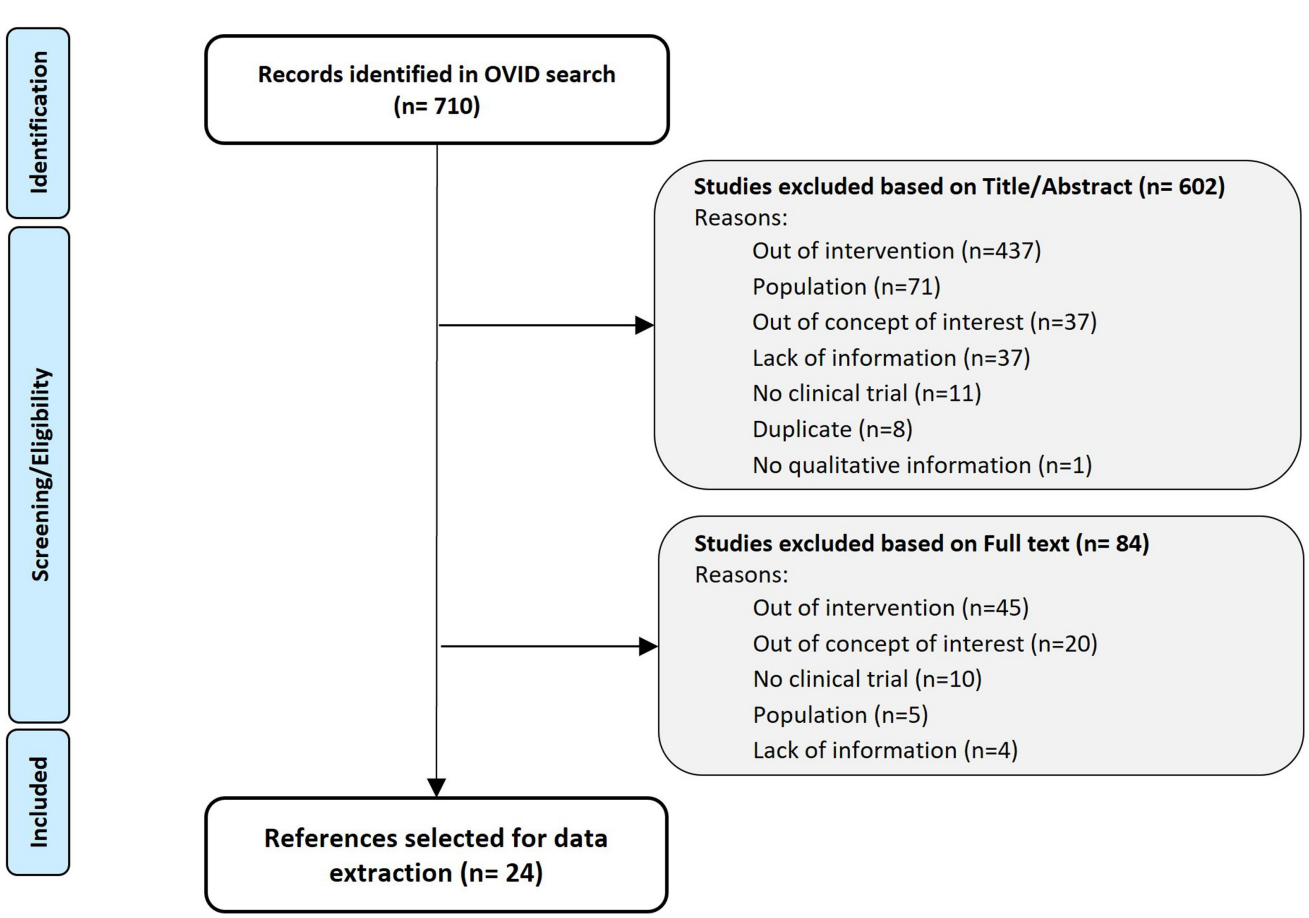
Targeted literature review PRISMA flow diagram (9).

Searches of in-trial interview studies published between January 2011 and February 2022 was conducted in Medline and Embase databases (via OVID platform) using Thesaurus, MeSH, and free text terms (See Search Strategy in Table 1). The search retrieved 710 references that were reviewed and selected according to the PICOS criteria (8). Although specific therapeutic indications were initially included, the selection was extended to chronic and non-oncologic diseases if the outcome around the use and impact of in-trial interviews was discussed (Figure 2).

**Table 1:** Targeted literature review search strategy in Medline and Embase databases (via OVID platform) performed on 11th February, 2022.

Search#	Strategy
1	*Qualitative method-related terms*: exp. qualitative research/ OR exp. Interviews as Topic/ OR exp. Delphi Technique OR exp. Anthropology, Cultural/ OR exp. Concept Formation/ AND (patient* interview* or interview* of patient* or exit interview* or in-depth interview* or semi-structured interview* or semistructured interview* or longitudinal interview* or entry interview* or Longitudinal qualitative research* or Longitudinal qualitative metod*OR mixed method* research* or mixed-method* research* or mixed method* research* or mixed-method* research* or mixed-methods stud* or mixed methods stud* or mixed methodology* or thematic analysis or thematic approach or embed* interview* or nested qualitative or ancillary qualitative or embed* qualitative or mid-point interview* or group-concept mapping or ethnographic).ab,ti.
2	*Clinical trial-related terms*: exp. clinical trial/ OR (clinical trial* or clinical stud*).ab,ti
3	*Drug-related terms*: (drug* or treatmen* or intervention*).ab,ti.
4	*Indication/Concept-related terms*: exp. Multiple Sclerosis/ OR exp. Lupus Erythematosus, Systemic/ OR exp. Myositis/ OR exp. “Squamous Cell Carcinoma of Head and Neck”/ OR exp. Carcinoma, Non-Small-Cell Lung/ OR exp. Small Cell Lung Carcinoma/ OR exp. Uterine Cervical Neoplasms/ OR exp. Lupus Erythematosus, Cutaneous/ OR exp. Lupus Nephritis/ OR exp. Arthritis, Rheumatoid/ OR exp. Dermatitis, Atopic/ OR exp. Esophageal Neoplasms/ OR exp. Neoplasms/ OR exp. Immune System diseases/ OR exp. Skin diseases/ OR exp. Nervous System Diseases/ OR exp. Musculoskeletal Diseases/ OR (Multiple sclerosis or Lupus Erythematosus or Libman-Sacks Disease or Myositis or Myopathy or Myopathies or Inflammatory Muscle Diseases or Squamous Cell Carcinoma of Head and Neck or HNSCC or Hypopharyngeal Squamous Cell Carcinoma or Laryngeal Squamous Cell Carcinoma or Oral Cavity Squamous Cell Carcinoma or Oral Squamous Cell Carcinoma or Oral Tongue Squamous Cell Carcinoma or Oropharyngeal Squamous Cell Carcinoma or Squamous Cell Carcinoma of Larynx or Squamous Cell Carcinoma of the Mouth or Squamous Cell Carcinoma of the Nasal Cavity or NSCLC or Non-Small Cell Lung Cancer or Non-Small Cell Lung Carcinoma or Non-Small-Cell Lung Carcinoma or Non-small Cell Lung Cancer or Small Cell Lung Carcinoma or SCLC or Oat Cell Carcinoma of Lung or Oat Cell Lung Cancer or Small Cell Cancer Of The Lung or Small Cell Lung Cancer or Uterine Cervical Neoplasms or Cancer of Cervix or Cancer of the Uterine Cervix or Cervical Cancer or Cervical Neoplasms or Cervix Cancer or Cervix Neoplasms or Uterine Cervical Cancer or Glomerulonephritis or Rheumatoid Arthritis or Neurodermatitis or eczema or Esophag* Cancer or Esophag* Neoplasm or Optic neurositis OR patient* perception* or patient* experience).ab,ti.
5	#1 AND #2 AND #3 AND #4 AND English, Abstract, from January 2011 to February 2022

Search in Mesh Tree (Medline) thesaurus (Embase) and exploded (exp); unlimited truncation (*): any word that begins with the term will be retrieved; search in abstract and title (ab.ti).

**Figure 3:**
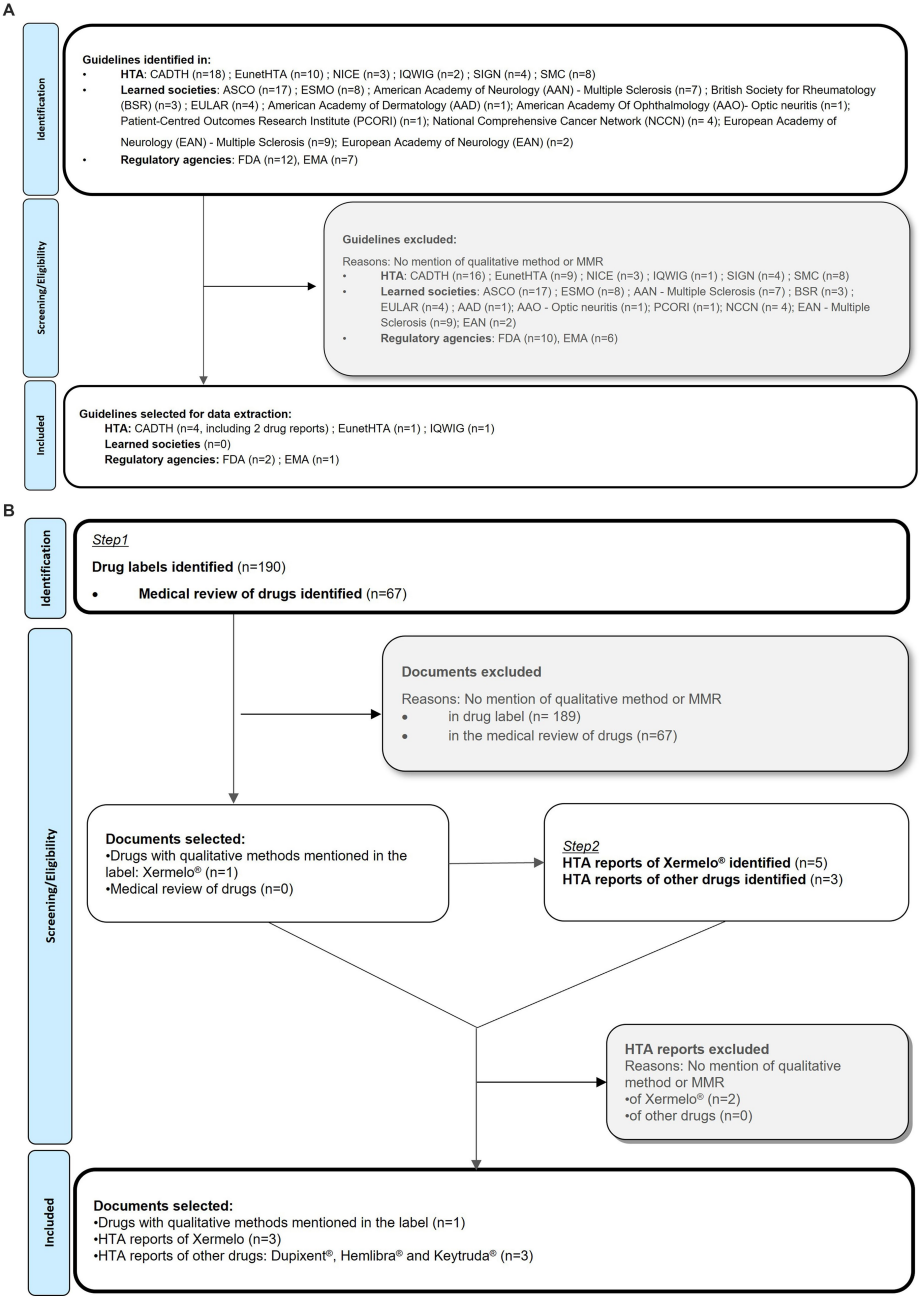
**(A)** Grey literature review of guidelines and documents mentioning in-trail interviews PRISMA flow diagram (9). **(B)** Grey literature review of drug labels, drug reports and HTA reports PRISMA flow diagram.

Searches for guidelines or documents issued by regulatory agencies as of February 2022, as well as reports from health technology agencies (HTA) or learned societies was performed. We sought to find documents which included discussion and recommendations on the use and impact of qualitative research in clinical trial settings. The searches were performed in the PROINSIGHT™ database (10) and the websites of HTA, regulatory agencies and learned societies (Table 2). This search identified 19 documents issued by regulatory agencies, 45 documents from HTAs and 57 from learned societies. The documents (including drug reports and guidelines) were reviewed and selected if they discussed or included recommendations around the use and impact of qualitative studies, in a clinical trial context. Documents with no qualitative method research information, about PROs development or not related to the context of drug development were excluded (Figure 3A).

**Table 2:** Grey literature review search strategy performed on 22nd February, 2022.

Search#	Strategy
1	Sources
	*Databases*: Mapi Research Trust PROLABELS™ OR PROINSIGHT™
	*Websites*: Food and Drug Administration (FDA) OR European Medicine Agency (EMA) OR Canadian Agency for Drugs & Technologies in Health (CADTH) OR Pharmaceutical Benefits Advisory Committee (PBAC) OR National Institute for health and care excellence (NICE) OR Scottish Intercollegiate Guidelines Network (SIGN) OR Scottish Medicines Consortium (SMC) OR Institut für Qualität und Wirtschaftlichkeit im Gesundheitswesen (IQWIG) OR European Network for Health Technology Assessment (EunetHTA) OR National Comprehensive Cancer Network (NCCN) OR European Society for Medical Oncology (ESMO) OR American Society of Clinical Oncology (ASCO) OR American Academy of Dermatology (AAD) OR European Academy of Dermatology and Venereology (EADV) OR American Academy of Neurology OR American Academy of Neurology (AAN) OR European Alliance of Associations for Rheumatology (EULAR) OR British Society for Rheumatology (BSR) OR American Academy of Ophthalmology (AAO)
2	*MMR OR qualitative method*: interview OR preference OR satisfaction OR qualitative OR patient experience OR patient’s experience
3	*Document*: guideline OR label OR drug report
4	*Indication*: Multiple sclerosis or Lupus Erythematosus or Libman-Sacks Disease or Myositis or Myopathy or Squamous Cell Carcinoma of Head and Neck or HNSCC or Hypopharyngeal Squamous Cell Carcinoma or Laryngeal Squamous Cell Carcinoma or Oral Cavity Squamous Cell Carcinoma or Oral Squamous Cell Carcinoma or Oral Tongue Squamous Cell Carcinoma or Oropharyngeal Squamous Cell Carcinoma or Squamous Cell Carcinoma of Larynx or Squamous Cell Carcinoma of the Mouth or Squamous Cell Carcinoma of the Nasal Cavity or NSCLC or Non-Small Cell Lung Cancer or Non-Small Cell Lung Carcinoma or Small Cell Lung Carcinoma or SCLC or Oat Cell Carcinoma of Lung or Oat Cell Lung Cancer or Small Cell Cancer Of The Lung or Small Cell Lung Cancer or Uterine Cervical Neoplasms or Cancer of Cervix or Cancer of the Uterine Cervix or Cervical Cancer or Cervical Neoplasms or Cervix Cancer or Cervix Neoplasms or Uterine Cervical Cancer or Glomerulonephritis or Rheumatoid Arthritis or Neurodermatitis or eczema
5	#1 AND #2 AND #3 AND #4

Finally, a search in the PROLABELS™ database was performed in February 2022, to find any examples of inclusion of evidence, data or results from interviews in product labeling. PROLABELS™ is a database on PRO endpoint strategies used to demonstrate the efficacy and safety of drugs or devices in the market approval (11). The search used free text terms related to qualitative methods or concepts and retrieved 257 labels and medical reviews (Table 2). The labels were reviewed and selected if they mentioned the use of qualitative methods (Figure 3B). HTA reports were subsequently searched to check if qualitative methods were used for reimbursement evaluation by payers.

## 3. Results

Twenty-four articles, including 22 peer-reviewed articles and two conference abstracts on in-trial interview studies were extracted; seven guidelines gave recommendations about the use of patient input and qualitative research in clinical trials; one drug label, two drug reports and four HTA reports showed how qualitative interviews have been used by health authorities (Figure 1).

### 3.1. Peer-reviewed articles review

The 27 studies described in the 24 articles reviewed addressed different research questions, and were based on different clinical populations, method, study design, and settings (Table 3). The research questions included: patients clinical trial experience (*n* = 7), treatment satisfaction and acceptability (*n* = 7), treatment adherence (*n* = 4), change in quality of life (QoL) (*n* = 4), assessment of signs and symptoms (*n* = 3), intervention success (*n* = 3), treatment benefit (*n* = 2), health outcomes (*n* = 1), coping strategies (*n* = 1), social functioning (*n* = 1), decision making process (*n* = 1) and treatment experience (*n* = 1). More than one research question was addressed in some studies, for example one study explored signs and symptoms and the change in QoL (28) while another explored signs and symptoms, clinical trial experience and treatment satisfaction/acceptability (29). In most studies (*n* = 18), interviewees included patients only; five studies included interviews with patients, parents, partners and/or a clinician, one with community members, and three studies did not mention the population interviewed. The number of interviewees ranged from 9 to 78. The therapeutic areas in which studies were conducted included oncology (*n* = 7), infectious diseases (*n* = 7), respiratory tract diseases (*n* = 3), digestive system diseases (*n* = 2), cardiovascular diseases (*n* = 2), nervous system diseases (*n* = 1), mental health disorders (*n* = 1) and eye disease (*n* = 1; Table 3). Several qualitative data collection methods were described with one-on-one interviews being the most common (*n* = 26). Interviews were semi-structured (*n* = 16), in-depth (*n* = 8), serial ethnographic interviews (*n* = 1), or key informant interviews (*n* = 1). Other qualitative methods included focus groups (*n* = 5) and direct observations of participants and processes as a complement to interviews (*n* = 2). Three studies mentioned the inclusion of interviews but did not specify a particular type of interviews. Interviews were conducted using different modes, including telephone (*n* = 9), face-to-face (*n* = 7), both face-to-face and by telephone/video call (*n* = 2); nine studies did not specify the mode used. Face-to-face interviews were conducted either at the interviewee’s home or on site (hospital). Interviewers were from qualitative (*n* = 13), social science (*n* = 2), medical (*n* = 1), or local trial staff (*n* = 1) backgrounds; ten studies did not specify the professional background of the interviewers. Among the studies retrieved, 11 followed a longitudinal design with entry/baseline and/or intermediate and/or exit interviews (12, 22–26, 29, 30, 33, 36, 37), seven were exit interviews only (13, 16, 21, 28, 31, 34, 35), two were entry interviews only (14, 26) and seven did not specify the interview time point. The interviews were embedded in phase 2 studies (*n* = 10), phase 2/3 (*n* = 1), phase 3 (*n* = 5), phase 4 (*n* = 1), in studies where the phase was not applicable (*n* = 7) or not specified (*n* = 3). Participants interviewed included the entire trial population or a subpopulation. The interviews were conducted with all study participants in three trials (14, 26, 32) and with a subset of the study participants (median of about 14%) in 20 trials (12, 13, 15–18, 21–25, 27–31, 33–36).

**Table 3:** Summary table of the characteristics of the studies retrieved by the literature review (*n* = 24 articles, *n* = 27 studies).

Research question	Therapeutic indication	Number of Interviewees	Trial design	Phase	Reference
Adherence	HIV	Patients (*n* = 54; total in trial = 2,120; total in arm = 180)	- Randomized, double-blind, placebo-controlled trial	phase 3	Agot et al. (12)
			- Embedded qualitative study		
	HIV	Patients (*n* = 60; total in trial = 179)	- Randomized, open-label clinical trial	phase 2	Amico et al. (2, 13)
			- Qualitative sub-study		
	HIV	Patients (*n* = 63)	- Community-level cluster randomized HIV test-and-treat trial	Phase N/A	Ayieko et al. (14)
			- Embedded qualitative study		
	HIV	Patients (*n* = 102; total in trial = 5,029*), partners (*n* = 22), community advisory board members (*n* = 17), community stakeholders (*n* = 23)	- Double-blind, five-arm randomized, placebo-controlled pre-exposure prophylaxis (PrEP) trial	phase 2b	Van Der Straten et al. (15)
			- Qualitative exploratory ancillary study		
Change in QoL	Major depressive disorder	Patients (*n* = 23; total in trial = 1,148*)	- Open-label, long-term extension safety trial	phase 3	Starr et al. (16)
Change in QoL Treatment experience	Recurrent pericarditis	Patients (*n* = 10; total in trial = 26*)	- Multicenter, open-label, single-active arm trial	phase 2	Lin et al. (17)
Clinical trial experience	Bladder cancer	Patients (*n* = 24; total in trial = 45)	- Non-inferiority randomized clinical trial	phase 3	Moynihan et al. (18)
			- Embedded feasibility stage and qualitative study		
	Breast cancer	Not specified	- ZICE randomized clinical trial	phase 3*	Nelson et al. (19, 20)
			- Multicentre, qualitative sub-study* (QualZICE)		
	Chronic or refractory breathlessness	Patients [*n* = 22; total in trial = 64 (isrctn.com)]	- Double-blind randomized trial	Phase N/A	Lovell et al. (21)
			- Embedded qualitative study		
	HIV-associated cryptococcal meningitis	Patients (*n* = up to 60; total in trial = 850), next of kin (*n* = up to 45) and researchers (*n* = up to 56)	- Multicentred randomized controlled trial	phase 3	Lawrence et al. (22)
	Prostate cancer	Patients (*n* = 15; total in trial = 201*)	- Multisite randomized, non-blinded trial	phase 2	Viljoen et al. (23)
			- Nested qualitative study (QualTheraP)		
Clinical trial experience change in QoL	Gastrointestinal stromal tumor (GIST)	Patients (*n* = 9; total in trial = 31*)	- Prospective, open-label, 1-group, multicenter trial	phase 2	Fauske et al. (24)
Copings social functioning	Barth syndrome	Patients (*n* = 11; total in trial = 11) and parents (*n* = 8)	- Single-center, double-blinded, randomized, placebo-controlled crossover study	phase 2	Searle et al. (25)
Intervention success	HIV	Not specified	- Interventional, randomized, parallel assignment, open label trial*	Phase N/A	Camlin et al. (26)
Intervention success health outcomes	HIV	Community members, community leaders and healthcare providers, participant observation at community health campaigns (CHCs) and CHC attendees	- Interventional, community based, randomized, parallel assignment, open label trial*	Phase N/A	Camlin et al. (26)
	HIV	Not specified	- Interventional, randomized, crossover assignment, open label trial*	Phase N/A	Camlin et al. (26)
Intervention success Treatment satisfaction/acceptability	HIV	Children and young people (aged 8 to 24) living with HIV	- Open-label, randomized, parallel group non-inferiority trial [from The BREATHER (PENTA 16) Trial Group 2016]	phase 2/3	Camlin et al. (26)
Signs and symptoms	Carcinoid syndrome	Patients (*n* = 11; total in trial = 23*)	- Multicenter, randomized, double-blind, placebo-controlled, ascending, multidose study*	phase 2	Gelhorn et al. (27)
Signs and symptoms change in QoL	Multiple sclerosis	Patients (*n* = 18; total in trial = 31) and partners	- Double-blind, placebo controlled, randomized controlled, crossover trial	Phase N/A	Khan et al. (28)
			- Embedded qualitative study		
Signs and symptoms clinical trial experience treatment satisfaction/acceptability	Diabetic gastroparesis	Patients (*n* = 78; total in trial = 90)	- Placebo-controlled, double-blinded trial	phase 2	Ervin et al. (29)
			- Exit interviews		
Treatment benefit	Merkel cell carcinoma	Patients (*n* = 9; total in trial = 88)	- Convergent mixed methods research*	phase 2	Bharmal et al. (30)
Treatment experience decision making process	Endometrial cancer	Patients (*n* = 21; total at site = 96)	- Open label, three-arm randomized trial	Phase 2	O’Hara et al. (31)
Treatment satisfaction/acceptability	Geographic atrophy	Patients (*n* = 30)	- Mixed-methods cross-sectional study	Not specified	Enoch et al. (32)
	HIV and malaria co-infection	Patients (*n* = 114; total in trial = 830*) and HCP (*n* = 10)	- Qualitative study was conducted alongside a clinical controlled study*	phase 4	Mangesho et al. (33)
	Mild asthma	Patients (*n* = 35; total in trial = 675)	- Open-label, parallel-group, multicentre, randomized controlled trial	phase 3	Foster et al. (34)
	Mild asthma	Patients (*n* = 35; total in trial = 675)	- Open-label randomized controlled trial	Not specified	Foster et al. (35)
	Ulcerative colitis	Patients (*n* = 20 first interview, *n* = 15 s interview; total in trial = 270)	- Two-arm pragmatic multi-center randomized controlled trial [isrctn.com]	Phase N/A	Rapport et al. 2019 (36)
			- Nested qualitative interview study		

* Information from ClinicalTrials.gov

### 3.2. Review of regulatory guidelines and documents mentioning in-trial interviews

Our searches for regulatory guidelines/documents highlighted the growing interest of regulators and HTAs in qualitative research throughout product development. Since 2013, seven guidelines [two from FDA (2, 38), one from EMA (39), one from the German agency IQWIG (40), two from the Canadian Agency CADTH (41, 42) and one from the European Network for HTA (EunetHTA)-EMA (43)] and two drug reports by the CADTH (44, 45) mentioning the use of qualitative interviews were issued. In its 2013 Guidance on “the Patient’s Voice in the Evaluation of Medicines,” the EMA acknowledged the importance of “consulting and involving patients” at both pre- and post- authorization stages (39). They commented that data from real-world settings could help understand the benefit–risk ratio of a medicine. At that time, EMA drafted a list of actions supporting patient involvement in the drug development journey including the need to identify where quantitative versus qualitative input is needed, and the development and validation of new tools for eliciting values and preferences and representing benefit and risk. In its 2016 Guidance on “Patient Preference Information,” the FDA supported the value of qualitative patient preference perspective in identifying outcomes most important to patients in the context of benefit–risk assessments, and in informing clinical trial design by participating in identifying endpoints that may be of greatest importance to patients (38). The FDA provided research questions that could be addressed by several qualitative methods, such as “how do people perceive this disease/this intervention?.” Additionally, using qualitative research in the earliest stages of the drug development can help frame the questions to be pursued in subsequent studies. The FDA’s “Patient-Focused Drug Development Guidance 2” dedicated a specific appendix (number 5) on in-trial interviews (2). In this guidance, the FDA described the potential usefulness of patient input gathered using in-trial interviews, including, for example, reporting changes in symptoms or functioning, insights on participants treatment expectations, anticipated and unanticipated symptoms and side-effects, and exploring viability of proposed dosing regimen. Recommendations include: conducting interviews before (i.e., screening/baseline interviews) or after (i.e., exit interviews) patients complete the main portion of the clinical study, ensuring a trained, neutral third-party interviewer to conduct the interview even if, in some instances, interviews can be conducted with next of kin (2) as they may have unique perspectives important to medical product development programs especially with children and confused or unconscious adults (22, 25). CADTH, in its 2020 guidance for “providing patient input” indicated that patient perspectives would improve the quality of CADTH reimbursement reviews. They stated, “the information that patient groups share with CADTH helps reviewers and expert review committee members understand the impact (both good and bad) that the treatment has on those taking it and on those caring for loved ones living with the disease” (42). The evidence presented in the reimbursement evaluation is mostly drawn from clinical and qualitative studies. CADTH discussed in 2022 the need to better understand which stage of the drug development, exploring the patient’s perspective would be the most useful in providing such evidence in its “CADTH framework for patient engagement in health technology assessment” guidance (41). IQWIG, in 2020 mentioned the use of MMR in its “general methods - version 6.0” document but did not provide further details (40). EunetHTA and EMA published a joint document in 2021 to guide applicants in the design, conduct and analysis of patient preference studies suitable to inform regulatory and reimbursement decision-making throughout the treatment development life cycle (43). The document discussed the use of qualitative methods, including interviews, in the context of preference studies.

Although we found examples in the literature of studies illustrating the guideline recommendations, these guidelines were not mentioned in any of the articles retrieved from the literature review.

### 3.3. Case studies–telotristat ethyl, dupilumab, emicizumab and pembrolizumab

In addition to guidelines, we identified several examples demonstrating how evidence from qualitative interviews is used by health authorities. A search of EMA in the summary of product characteristics (SmPC), FDA’s labeling, and reports of major HTAs, on the use of qualitative interviews yielded four products: telotristat ethyl (Xermelo®), dupilumab (Dupixent®), emicizumab (Hemlibra®) and pembrolizumab (Keytruda®). The case of telotristat ethyl provides an understanding of what aspects of the qualitative evidence generated by the interviews have been considered by health authorities for market authorization and reimbursement. Telotristat ethyl is indicated in Malignant Carcinoid Syndrome (CS) and was approved by the FDA (February 2017) and by the EMA (September 2017). The drug developer conducted exit interviews on a subset of 35/135 patients participating in the pivotal TELESTAR study (46). In the SmPC, the EMA outlined that the patients exit interview sub-study was conducted over the telephone to assess “the relevance and clinical meaningfulness of symptom improvements […] and to further characterize the degree of change experienced during the trial” and satisfaction with telotristat ethyl. These exit interviews were not mentioned in the FDA label. The EMA public assessment report noted that a high percentage of patients (*n* = 21) reported high bowel movement frequency as the most important symptom of CS which impacted emotional, social and physical functioning. The interviews were also mentioned in the reports issued by the French Health Agency (HAS), All Wales Medicines Strategy Group (AWMSG) and CADTH to support telotristat ethyl reimbursement. The HAS agency expressed its inability to conclude on the results themselves due to the small sample size, which does not guarantee representativeness. The AWMSG, responsible for appraising new medicines for use in Wales considered the interviews as an added value for economic evaluation and a complement to the cost-utility measures captured in the quality-adjusted life year. Finally, the CADTH mentioned seven quotes from patients interviews to document their decision. The second case is that of dupilumab indicated for the treatment of moderate-to-severe atopic dermatitis. Dupilumab was approved by the FDA (March 2017) and by the EMA (October 2017). The drug developer conducted one-on-one interviews with study participants in Canada. The public assessment report from the CADTH summarized four key inputs from patients’ perspective. These interviews were not mentioned in the FDA label nor the EMA SmPC. The third case is emicizumab, which is indicated for routine prophylaxis of bleeding episodes in patients with hemophilia A. Emicizumab was approved by the FDA (November 2017) and the EMA (March 2018). The drug developer conducted one-on-one phone interviews with six health care professionals (HCP) involved in the phase III HAVEN program in France (three physicians, two nurses and one clinical research associate) (47). The public assessment report from the HAS mentioned only five HCP interviews and noted that the interviews were conducted because “the patient quality of life questionnaires used in the two-phase III studies did not demonstrate the impact of emicizumab on patients’ quality of life, as some investigators have suggested.” However, the data generated by the interviews were of an exploratory and not of a demonstrative nature. The interviews were mentioned in the 2018 public assessment report issued by the HAS but not in the 2019 report, nor in the FDA label and the EMA SmPC. The last case is pembrolizumab, which is indicated for the treatment of multiple cancers including the treatment of metastatic or unresectable recurrent head and neck squamous cell carcinoma. Pembrolizumab was first approved by the FDA (September 2014) and by the EMA (July 2015). The drug developer solicited patient input from a total of 13 respondents from the Life Saving Therapies Network patient group. The public assessment report from the CADTH focuses on one interviewee and two survey respondents; and they reported positive experiences of improvement of quality of life and ability to perform day-to-day tasks. Additionally, the CADTH noted that the three patients did not report any side effects associated with pembrolizumab and stated that it was effective in controlling their cancer compared to their previous treatment. Overall, the qualitative data informed the benefit–risk profile, with patients appreciating the increased efficacy of treatments, a better side effect profile, and improved quality of life. This was not mentioned in the FDA label nor in the EMA SmPC. In the four drug examples, regulators and HTA agencies focused their evaluation on the efficacy and safety of the drug and on the assessment of benefits and risks. The qualitative data generated by the interviews helped the regulators and HTA agencies to make positive decision about drugs approval and/or reimbursement.

## 4. Discussion

### 4.1. Use of qualitative interviews in the drug development

Combining qualitative research and clinical trials has the potential to generate additional evidence to support an investigational product. At the research or pre-clinical stage, qualitative methods, can be used as a novel, innovative approach to understand and describe the natural history of a disease and unmet medical needs. At the early stages of a drug’s clinical development, interviews are useful to inform on treatment benefit and safety profile. Indeed, patients reports on changes in the clinical manifestation of the disease may also indirectly inform the mechanism of action of the investigational drug. Additionally, when designing a phase 2 or 3 clinical trial, qualitative interviews should help determine the appropriate endpoints by providing insight into the most troublesome symptoms or aspects of functioning or quality of life that are most affected.

In-trial interviews allow for individualized, patient-centered insights of the risks and benefits of the experimental treatment and can be valuable to assess the viability of a proposed dosing regimen. Interviews can eventually support the identification, development, or adaptation of PRO measures for use in later phases or other trials in the same indication or population. Qualitative data is valuable in providing supportive evidence of the content validity of the primary or key secondary endpoints in health authorities submissions. When questions arise on the clinical meaningfulness of magnitude of change during regulatory or reimbursement review process, in-trial interviews may be critical in offering more granular data/information for the interpretation of such changes. After approval, evidence from interviews may help health authorities understand the benefit–risk ratio of a drug in a real-life context. Our literature review showed that in-trial interviews were mainly used in phase 2 or 3 trials. There was no indication, however, whether the studies were conducted for the purpose of a subsequent marketing authorization application.

Among the objectives described in the literature, eight (treatment satisfaction and acceptability, treatment adherence, change in quality of life, assessment of signs and symptoms, treatment benefit, health outcomes, social functioning, and treatment experience) could be of interest for the regulators and HTA agencies while evaluating the drug profile for market authorization or reimbursement. These objectives were explored in 21 of 27 studies (12–17, 24–36).

Regulatory and reimbursement guidance is needed to specify the information that would be most useful to regulators and HTA agencies in their assessment of the evidence that could be generated by qualitative studies embedded into clinical trials. A guideline would allow researchers to better define the objectives of their research, focus their efforts on certain assessments and provide evidence that meets the needs of agencies in their evaluation. Such guidance would complement what the FDA has initiated with its PFDD guidances (2, 48). This will enable greater certainty of decision-making by the authorities when it comes to getting a drug through the licensing and reimbursement process.

To make qualitative evidence more impactful, the objective of the qualitative interviews should be clearly defined and aligned with those of the clinical trial, and the questions asked to the participants should reflect these objectives. Crossing the data from the qualitative interviews and the clinical trial could generate new information on patients’ experience. It would be interesting to compare patients’ qualitative reports with clinical or biomarker or PRO measures. This approach could be valuable as it would involve the patients in triangulating their data, even if this design implies higher levels of data protection security for compliance with local regulations. In rare diseases (30), qualitative interviews are particularly relevant as the heterogeneity of the symptoms and their impacts on quality of life make it challenging to measure with a generic PRO questionnaire and there is often no possibility of investing in a disease-specific instrument. Whatever the objectives, therapeutic area or setting, to make in-trial interviews a successful outcome, communication and partnership between the clinical study team, the investigator sites, and the qualitative research team must be established as early as possible.

### 4.2. Methodological considerations

Regarding the methodology, the parameters for ensuring the quality and reproducibility of the results differ from those of quantitative research. Qualitative research aims at characterizing a phenomenon, this is why the sampling methods relies on the principle of saturation (the point where no new information seems to emerge from the data) (49, 50). Consequently, the number of interviews should be estimated before the interviews are conducted but may not be fixed. A subsample of the trial population may be interviewed (28), and it is recommended to pre-define the sampling method according to the objectives of the research (systematic, probability, purposive, convenience) to anticipate on reviewers’ comments as seen with telotristat. Interviewing all the clinical trial’s patients presents challenges which may be circumvented by achieving saturation in a smaller sample size (12, 13, 36). This aspect is often challenging to conceptualize for reviewers coming from a quantitative world and there is room for communication around this topic. Interviewing in qualitative research requires specific skills as the interview techniques have impacts on the result’s quality. The FDA generally recommends involving qualitatively trained, neutral third-party interviewers (2). Their role should be as neutral as possible, therefore choosing HCPs or study staff as interviewers is not recommended (2, 51, 52). Interviewees should be interviewed in local language to avoid cultural and language barriers. The mode of administration (in-person, telephone, online/virtual video conferences) and the setting of the interviews (individual interviews or focus groups) should be adapted to the population (29, 51).

A clinical trial can include one or more interviews at different time points. Timing considerations are directly linked to the research question (6) (Table 4). The FDA recommends conducting interviews before (screening/baseline interviews) or after (exit interviews) patients complete the main portion of the clinical study to avoid potential compromise of study integrity (2). Conducting interviews at entry mid-stage and exit of the same clinical trial allow for a longitudinal understanding of new therapies and their impact on patients’ daily life. The timing of the interviews should be discussed when qualitative research is designed. In exit interviews, especially for long-term clinical trials, recall bias is critical. Patients with chronic diseases may have difficulty describing the course of their disease before the trial and describing any changing experiences during the trial because of the complexity of their disease and the length of time since their symptoms began. In this case, interviewing patients at screening and then at several time points in the trial provides a qualitative baseline and can help manage recall bias. Longitudinal qualitative research yields a large amount of data given that the objective is to understand an experience over time. The complexity of longitudinal qualitative data makes the analysis challenging; selecting the analytical approach and developing a qualitative analysis plan are critical to ensure rigor and meet quality standards (53), and to ensure validity and reliability of the analysis. This also applies to cross-sectional analyzes conducted at trial entry or exit. It is important to note that since longitudinal qualitative research involves a larger number of interviews and analyzes, the cost is higher than that of a single interview. In-trial interviews should consider potential challenges. Regardless of what phase the trial is being conducted, the protocol, and informed consent form describing the interviews may be included in the trial protocol or may be developed separately (6). This information is not always described in the literature. The earlier in-trial interviews are envisaged in the design, the better as they can be included in the clinical protocol thereby avoiding additional amendments and ethics re-submissions. The content of the interview guides has a direct impact on the quality of the results. Hence, sufficient time should be allowed to work on interview objectives, so the resulting guide aligns with the research question and avoids being list or a survey of too many topics. Duration of the interview should consider the population interviewed. The format of the interview guide depends on the interviewer’s experience (thematic vs. narrative).

**Table 4:** Qualitative research within a clinical trial: considerations on time points and research questions.

	Screening/entry interviews	Intermediate	Exit interviews
Timing	• Participants are interviewed prior to the start of treatment	• Participants are interviewed while on treatment	• Participants are interviewed after treatment completion or at trial discontinuation
Examples of research questions:	• Understand patients’ experiences with their disease: ◦ The natural history and progression of the disease	• Understand treatment experience, disease progression and any critical events linked to the phenomenon of interest	• Understand perceptions and evaluations of treatment and trial, perception and meaning of treatment change, and potential pre-treatment comparisons• Understand the changes resulting from treatment, (especially when exit interviews are combined with screening interviews)
	◦ Most important/bothersome symptoms and their impacts on patients’ quality of life • Understand patients’ experience with previous treatment understand preferences or expectations for the new treatment	• Provide context and understanding of patients’ responses when the interviews are scheduled at the time patients’ complete patient-reported outcomes questionnaires	
Reference	Camlin and Seeley (26)	Rapport et al. (36)	Anthony et al. (46)
	Ayieko et al. (14)		

### 4.3. Operational considerations

Operationally, researchers must heed regulatory and data protection requirements, legal framework, ethics-related procedures, good clinical practices guidelines and pharmacovigilance. An example is the management of adverse events (AEs). Although AE reporting is not the purpose of the interviews, participants may spontaneously report them, as a result, management should anticipate how these reports will be managed as part of the operational set up of the trial.

For future considerations, an approach called Natural Language Processing (NLP) is emerging in health care research (9). NLP is a subfield of linguistics, computer science, and artificial intelligence that processes and analyzes large amounts of natural language data. NLP combined with in-trial interviews, would be a powerful strategy for analyzing and triangulating the information generated.

### 4.4. Limitations

Due to the nature of this literature review, various potential limitations are worth noting. The first relates to the search strategy; the search terms employed focused on specific therapeutic indications or areas, for example. The second limitation relates to the screening method used for study selection; a single researcher performed all screening. Given the purpose of this targeted, non-systematic literature review, an approach to use a single reviewer with extensive experience with literature reviews was considered appropriate. Despite these limitations, our approach ensured that we were able to address our main objective, i.e., to provide an understanding of the current use of qualitative interviews in drug development.

## 5. Conclusion

In-trial interviews is still emerging and not yet common practice. While there is increased interest in evidence generated *via* in-trial interviews throughout industry, scientific communities, regulatory agencies and HTAs, guidance from regulators and HTAs would be helpful and address what type of benchmark applies (e.g., good clinical practices). The scientific community could spearhead establishment of recommendations, best practice, and further development of methods/technologies to address common goals and challenges for such interviews. While acknowledging that qualitative research is not meant to replace quantitative research, and that it is a different and complementary valuable means of addressing questions during drug development, a mindset change within research and development in pharmaceutical industry is necessary to fully realize the potential of in-trial interviews as a tool for drug development.

## Author contributions

All authors listed have made a substantial, direct, and intellectual contribution to the work and approved it for publication.

## Funding

This study was funded by Merck KGaA. A-SM, AM, RA, CD-G, and SL are consultants paid by Merck for the conduct of the literature review and analysis. The authors declare that this study received funding from Merck KGaA. The funder was involved in the study design, analysis, interpretation of data, reviewing this article, and the decision to submit it for publication.

## Conflict of interest

A-SM, AM, RA, CD-G, and SL are employees of ICON. PS is an employee of LAIFE REPLY. PK is an employee of Merck KgaA. JP and ES are employees of EMD Serono (a company of Merck KgaA). ICON and LAIFE REPLY were contracted with Merck KgaA as consultants on patient-centered research activities.

## Publisher’s note

All claims expressed in this article are solely those of the authors and do not necessarily represent those of their affiliated organizations, or those of the publisher, the editors and the reviewers. Any product that may be evaluated in this article, or claim that may be made by its manufacturer, is not guaranteed or endorsed by the publisher.
